# Long-term increase in soluble interleukin-6 receptor levels in convalescents after mild COVID-19 infection

**DOI:** 10.3389/fimmu.2024.1488745

**Published:** 2025-01-06

**Authors:** Juliane Lokau, Yvonne Garbers, Manuel M. Vicente, Anna Dittrich, Stefan Meltendorf, Holger Lingel, Anja K. Münster-Kühnel, Monika Brunner-Weinzierl, Christoph Garbers

**Affiliations:** ^1^ Institute of Clinical Biochemistry, Hannover Medical School, Hannover, Germany; ^2^ Department of Pathology, Otto-von-Guericke-University Magdeburg, Medical Faculty, Magdeburg, Germany; ^3^ Faculty of Management, Culture and Technology (Lingen campus), Osnabrück University of Applied Sciences, Lingen, Germany; ^4^ Department of Systems Biology, Institute of Biology, Otto-von-Guericke-University Magdeburg, Magdeburg, Germany; ^5^ Department of Experimental Pediatrics, Otto-von-Guericke-University Magdeburg, Magdeburg, Germany

**Keywords:** interleukin-6, interleukin-6 receptor, gp130, COVID-19, sCD25

## Abstract

**Introduction:**

Serum levels of interleukin-6 (IL-6) are increased in COVID-19 patients. IL-6 is an effective therapeutic target in inflammatory diseases and tocilizumab, a monoclonal antibody that blocks signaling via the IL-6 receptor (IL-6R), is used to treat patients with severe COVID-19. However, the IL-6R exists in membrane-bound and soluble forms (sIL-6R), and the sIL-6R in combination with soluble glycoprotein 130 (sgp130) forms an IL-6-neutralizing buffer system capable of neutralizing small amounts of IL-6.

**Methods:**

In this study, we analyzed serum levels of IL-6, sIL-6R and sgp130 in the serum of COVID-19 convalescent individuals with a history of mild COVID-19 disease and in acute severely ill COVID-19 patients compared to uninfected control subjects. Furthermore, we used single cell RNA sequencing data in order to determine which immune cell types are sources and targets of the individual cytokines and whether their expression is altered in severe COVID-19 patients.

**Results:**

We find that sIL-6R levels are not only increased in acute severely ill patients, but also in convalescents after a mild COVID-19 infection. We show that this increase in sIL-6R results in an enhanced capacity of the sIL-6R/sgp130 buffer system, but that significantly enhanced free IL-6 is still present due to an overload of the buffer. Further, we identify IL-6 serum levels, age and the number of known pre-existing medical conditions as crucial determinants of disease outcome for the patients. We also show that IL-11 has no major systemic role in COVID-19 patients and that sCD25 is only increased in acute severely ill COVID-19 patients, but not in mild convalescent individuals.

**Discussion:**

In conclusion, our study shows long-lasting alterations of the IL-6 system after COVID-19 disease, which might be relevant when applying anti-IL-6 or anti-IL-6R therapy.

## Introduction

Coronavirus disease 2019 (COVID-19) is an infectious disease caused by the severe acute respiratory syndrome coronavirus 2 (SARS-CoV-2) that originated from Wuhan, Hubei Province, China, in late 2019 (1). Declared a worldwide pandemic by the WHO shortly afterwards, COVID-19 has caused more than 6 million deaths worldwide to date. Despite serious efforts to prevent spreading of the virus, including vaccination, temporarily closing of restaurants and businesses, increased abilities to work from home, enforced reduction of social contacts and mandatory wearing of face masks, infection rates have been high most of the time since the beginning of the pandemic, at least in part due to constant evolution of the virus (2).

Several risk factors are known to influence the morbidity and mortality of the COVID-19 disease. Among them are male sex, age of the patients (3–5), smoking (6, 7), being overweight (8) and pre-existing medical conditions like hypertension or type 2 diabetes (6, 9) among others.

Cytokines are small, secreted proteins that play critical roles in health and disease. A typical hallmark of COVID-19 is the production and release of several pro-inflammatory cytokines, which help to sustain the inflammation and also contribute to recruiting different immune cell types towards the lung (10). Excessive production of such pro-inflammatory cytokines can result in a hyperinflammatory syndrome, which is reminiscent of e.g. the cytokine storm in patients undergoing CAR-T-cell therapy, and associated with death of the patients (11). One of these pro-inflammatory cytokines is interleukin-6 (IL-6), the name-giving member of the IL-6 family of cytokines (12). Other family members are IL-11, ciliary neurotrophic factor (CNTF), leukemia inhibitory factor (LIF), oncostatin M (OSM), cardiotrophin-1 (CT-1), cardiotrophin-like cytokine (CLC), IL-27 and IL-31 (12). With the exception of IL-31, they use the β-receptor glycoprotein 130 (gp130) to activate intracellular signaling cascades in their target cells, most notably the Janus kinase/signal transducer and activator of transcription (Jak/STAT) pathway (13). While most of the family members can bind to and activate their β-receptors directly, IL-6 and IL-11 have to bind first to unique non-signaling α-receptors on their target cells, which are termed IL-6 receptor (IL-6R) and IL-11R, respectively (14). The resulting IL-6/IL-6R and IL-11/IL-11R complexes then recruit a gp130 homodimer and induce signal transduction. Due to the ubiquitous expression of gp130, the expression patterns of IL-6R and IL-11R determine which cells respond to the cytokines and which do not. Signaling via membrane-bound IL-6R and IL-11R has been termed classic signaling. In addition, soluble forms of both receptors have been described which bind their ligands with the same affinity as their membrane-tethered counterparts, and the sIL-6R/IL-6 and sIL-11R/IL-11 complexes can bind to and activate gp130 homodimers equally well (termed trans-signaling), thereby significantly widening the spectrum of cells that can be activated by these cytokines (15–17). The major mechanism to generate sIL-6R is proteolytic cleavage of the membrane-bound precursor by the metalloproteases ADAM10 and ADAM17, while alternative splicing of the *IL6R* mRNA, which generates sIL-6R via excision of the exon encoding the transmembrane region, only accounts for up to 20% of sIL-6R (18). sIL-11R appears to be generated exclusively by proteolysis, and ADAM10 and RHBDL2 have been identified so far as responsible proteases (19, 20). Serum levels of sIL-6R in healthy humans are usually in the range of 20–70 ng/ml (21), while serum levels of sIL-11R are lower (19). Furthermore, also soluble forms of gp130 (sgp130) exist, which are generated by both alternative splicing and proteolytic cleavage (22–24), predominantly by the protease BACE1 (25). Sgp130 levels in human serum are usually in the range of 400 ng/ml (17). The functional roles of these soluble cytokine receptors are still under investigation, but recent studies have provided evidence that sIL-6R and sgp130 together form a sIL-6R/sgp130 buffer, which binds and thus eliminates low levels of circulating IL-6, thereby counteracting low grade inflammation (24, 26, 27). Importantly, soluble cytokine receptors exist not only within the IL-6 family (28). We have recently shown that soluble IL-2Rα/CD25 (sIL-2Rα/sCD25) is also generated by proteolysis through ADAM10 and ADAM17 and able to modulate IL-2 signaling in T cells (29, 30).

IL-6 has important roles in tissue homeostasis and immune responses, as it is e.g. crucial for regeneration of the gut epithelium, regeneration of the liver after injury or the differentiation and proliferation of different T cell subsets [reviewed in (31–33)]. Furthermore, it contributes to numerous inflammatory diseases and is an important therapeutic target (31, 34). Several antibodies are used in the clinics that either target IL-6 or the IL-6R with tocilizumab, which targets the cytokine-binding site of the IL-6R, as the most prominent example (34). The next generation of IL-6-blocking therapeutics that selectively block only the trans-signaling pathway, are currently in clinical studies (17, 35, 36). Given that IL-6 levels in the serum are predictors of COVID-19 severity (37), it is not surprising that tocilizumab is used to treat COVID-19 patients (38–41). We have previously shown that not only IL-6 is important in this regard, but rather that e.g. high levels of IL-6, sIL-6R and sgp130 are independent predictors of COVID-19 severity in survivor patients, whereas e.g. high levels of IL-6 and low levels of sIL-6R and sgp130 were predictors of death in a subgroup of patients with a very poor prognosis (42).

In the present study, we analyzed IL-6, IL-11, sIL-6R, sgp130 and sCD25 in the serum of healthy, uninfected subjects, in COVID-19 convalescent individuals with a history of mild COVID-19 disease and in acute severely ill COVID-19 patients. We used single cell RNA sequencing data in order to determine which immune cell types are sources and targets of the individual cytokines and whether their expression differs between the groups. Further, we identify confounding factors that contribute to differences between healthy and sick individuals.

## Materials and methods

### Study design and subjects

Serum samples of 49 healthy individuals who were not previously exposed to SARS-CoV-2 (judged from no detectable symptoms and no presence of SARS-CoV2 RNA or anti-SARS-CoV-2 antibodies) were analyzed and compared to sera from 68 convalescent individuals after a previous mild COVID-19 disease (mild symptoms that did not require hospitalization during the acute disease) and 25 acutely ill COVID-19 patients with severe symptoms that were treated at the ICU at the time of blood sampling. The convalescent and the healthy individuals were examined from April to November 2020 (acquired with less than 10 infected persons per 100,000 inhabitants). The acutely ill COVID-19 patients were examined in December 2020 and January 2021. All further details on the study design and the participating patients can be found in previous publications (43, 44). All samples were collected before vaccination against COVID-19 was available. Characteristics of the three patients groups are also given in **Table 1**, and the cytokine profile of the three groups is given in **Table 2**.

**Table 1:** Characteristics of the study population.

	HD	MC	ICU
Participants (n)	49	68	25
Age (years)	47.4 ± 2.4	48.2 ± 1.9	66.4 ± 2.4
Gender, male, n (%)	16 (32.7)	28 (41.2)	15 (60)
BMI (kg/m²)	24.8 ± 0.6	26.3 ± 0.6^§^	26.7 ± 1.1

Data of the participants are shown as mean ± SEM. HD, healthy unexposed; MC, mild COVID-19 convalescent; ICU, acute severe COVID-19; BMI, body mass index. ^§^information regarding BMI was only available for 65 patients.

**Table 2:** Serum profiles of the study participants.

Serum protein	HD	MC	ICU
IL-6 sIL-6R sgp130 IL-11 sCD25	14.7 ± 1.3 (0.6 ± 0.06) 11.0 ± 1.2 (291.4 ± 24.8) 252.1 ± 5.6 (2,525 ± 56.5) 389.3 ± 151.5 854.1 ± 82.7	21.9 ± 2.1 (0.9 ± 0.09) 17.2 ± 1.5 (344.1 ± 29.3) 248.7 ± 6.2 (2,492 ± 62.4) 210.7 ± 58.8 765.4 ± 43.0	186.1 ± 67.9 (7.9 ± 2.9) 26.2 ± 3.9 (523.8 ± 77.8) 267.6 ± 9.8 (2,681 ± 98.4) 161.7 ± 91.6 4,171.0 ± 793.5

Serum levels of IL-6 (pg/ml), soluble interleukin-6 receptor (sIL-6R, ng/ml) and soluble gp130 (sgp130, ng/ml), IL-11 (pg/ml) and sCD25 (pg/ml) in healthy unexposed (HD), mild COVID-19 convalescent (MC) and acute severe COVID-19 patients (ICU). Values for IL-6, sIL-6R and sgp130 are additionally shown in pM in brackets. Serum levels are shown as mean ± SEM.

### Enzyme-linked immunosorbent assays

For the detection of sIL-6R, sgp130, IL-11, and sCD25 in human serum, DuoSet ELISA Kits (R&D System) were used according to manufacturers’ instructions. Where necessary, samples were diluted to stay within the detection range of the ELISA kit. The detection limits were as follows: 31.2 pg/ml IL-11, 6.2 ng/ml sIL-6R, 62.4 ng/ml sgp130, and 7.8 pg/ml sCD25. IL-6 serum levels have been published previously (43).

### Statistical analysis

Statistical analyses were performed using IBM SPSS Statistics (Version 29). Normal distribution was evaluated by the Shapiro-Wilk test and the Kolmogorow-Smirnow test. Spearman rank correlation tests were used to evaluate the correlations between protein serum levels, BMI, age, sex and the number of known pre-existing conditions. One-way analyses of variances (ANOVA) for all five serum proteins were applied. Consecutively, Tukey’s multiple comparison tests (i.e., test of contrast-coefficients) as *post hoc* analyses were used to test for differences between healthy control participants and different groups of patients. Multiple contrast-tests are superior to ANOVA and mean comparisons with t-Tests in two samples regarding power and information (45). All p-values are two-tailed, and a p-value below 0.05 was considered as statistically significant.

### Calculation of IL-6:sIL-6R and IL-6:sIL-6R:sgp130 complexes

The mass action law was applied to estimate the concentrations of the dimer of IL-6 and sIL-6R and the trimer of IL-6, sIL-6R and sgp130 in serum. First, based on the measured amounts of IL-6 and sIL-6R, the concentration of the IL-6:sIL-6R dimer was calculated. In the next step, based on the result of this calculation and the measured concentration of sgp130, the concentration of the IL-6:sIL-6R:sgp130 trimer was calculated.

The equilibrium concentration of IL-6, sIL-6R and IL-6:sIL-6R dimer is described by Equation 1,


(1) $${\text{K}}_{\text{D}1}\text{ = }\frac{\left(\left[\text{IL}-6\right]-\left[\text{Dimer}\right]\right)\left(\;\left[\text{sIL}-6\text{R}\right]-\left[\text{Dimer}\right]\right)}{\left[\text{Dimer}\right]}$$

To calculate the concentration IL-6:sIL-6R dimers, Equation 1 was rearranged to [Dimer], which results in Equation 2,


(2) $$\begin{array}{c} \left[\text{Dimer}\right]\;=\;0.5\;(\left[\text{IL}-6\right]+\left[\text{sIL}-6\text{R}\right]{\;+\text{ K}}_{\text{D}1})\;\\ -\sqrt{{\left(\frac{\left[\text{IL}-6\right]\;+\;\left[\text{sIL}-6\text{R}\right]{\;+\text{ K}}_{\text{D}1}}{2}\right)}^{2}\text{- }\left[\text{IL}-6\right]\left[\text{sIL}-6\text{R}\right]} \end{array}$$

Assuming that one sgp130 protein binds to one IL-6:sIL-6R dimer, the concentration of the IL-6:sIL-6R:sgp130 trimer was calculated accordingly using Equation 3.


(3) $$\begin{array}{c} \left[\text{Trimer}\right]\;=\;0.5\;(\left[\text{Dimer}\right]+\left[\text{sgp}130\right]{+\text{ K}}_{\text{D}2})\;\\ -\sqrt{{\left(\frac{\left[\text{Dimer}\right]\;+\;\left[\text{sgp}130\right]{\;+\text{ K}}_{\text{D}2}}{2}\right)}^{2}-\;\left[\text{Dimer}\right]\left[\text{sgp}130\right]} \end{array}$$

Molar concentrations of IL-6 (23.7 kDa), sIL-6R (50 kDa), and sgp130 (100 kDa) in serum were calculated for each patient based on the molecular weights of the three proteins. The dissociation constants of the IL-6:sIL-6R dimer and the IL-6: sIL-6R:sgp130 trimer are K_D1_ = 0.5 nM (46) and K_D2_ = 0.05 nM (47, 48), respectively.

### Single-cell RNA sequencing data processing

The publicly available single-cell RNA sequencing dataset of PBMCs from COVID-19 patients and controls (49) was downloaded from the fastgenomics repository (Schulte-Schrepping_2020_COVID19_10x_PBMC dataset, as.h5ad file). Quality control was performed using *scanpy* version 1.7.2 (50), which included filtering out cells with fewer than 200 genes detected and genes expressed in fewer than 3 cells. Clustering analysis and cell type assignment information were used from the dataset. The expression levels for genes of interest were interrogated and visualized using *scanpy*’s built-in plotting functions. Differential gene expression analysis was done by normalizing the total raw counts per cell, log transforming the data and performing the comparison between “control” and “severe” groups, for specific cell types, using *scanpy*’s built-in functions.

## Results

### IL-6 and sIL-6R, but not sgp130 serum levels, are increased in acute severe COVID-19 patients

**Figure 1:**
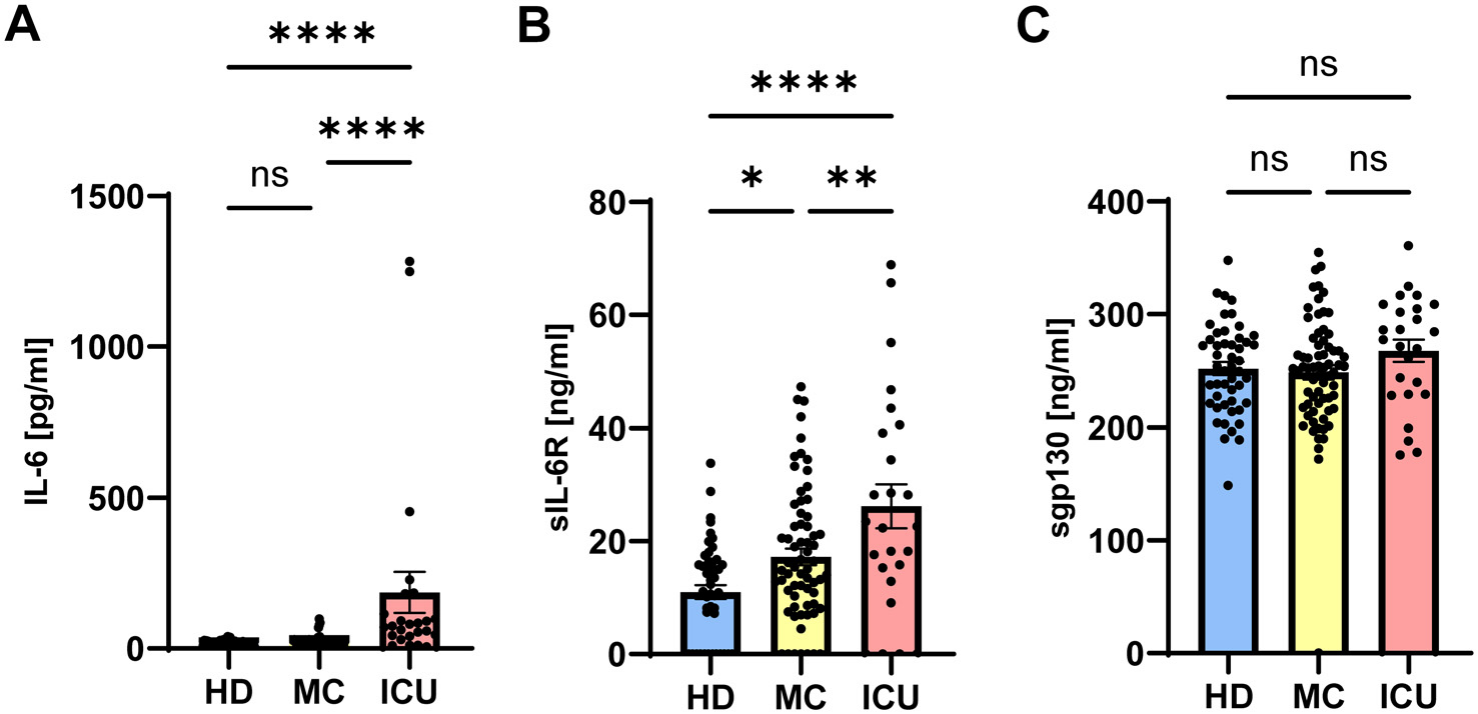
Serum levels of sIL-6R are increased in mild COVID-19 convalescent and acute severe COVID-19 patients. **(A–C)** Levels of **(A)** IL-6, **(B)** sIL-6R and **(C)** sgp130 were determined by ELISA in serum samples of 49 healthy unexposed individuals (HD), 68 mild COVID-19 convalescent (MC) and 25 acute severe COVID-19 patients (ICU). Serum amounts of each individual are shown as dots. The mean is indicated by bar graph, and the error bars denote SEM. Data were analyzed using one-way ANOVA followed by Tukey *post-hoc* test. The p values are shown above the respective diagrams as follows: *p < 0.05; **p < 0.01; ****p < 0.0001. n.s. denotes no significant difference.

The activity of IL-6 is controlled by sIL-6R and sgp130 proteins, which are present in rather high amounts in human blood. The amounts of sIL-6R and sgp130 remain mostly constant, even in patients with inflammatory diseases (51). In contrast, only few pg/ml IL-6 can be detected in healthy individuals, but these amounts can rise by several orders of magnitude during inflammation and infection (24). We have previously reported that the balance between sIL-6R and sgp130, which form a buffer system to neutralize small amounts of systemic IL-6, is disturbed in type 2 diabetes patients (27). We have additionally shown that such alterations occur also in patients with severe COVID-19 infections (42). In order to investigate whether such a phenomenon is also present in mild convalescent COVID-19 patients, we quantified IL-6, sIL-6R and sgp130 via ELISA in serum samples from 49 healthy unexposed individuals (HD), 68 mild COVID-19 convalescents (MC) and 25 acute severe COVID-19 patients (ICU, **Table 1**). As shown in **Figure 1A**, IL-6 levels were expectedly low in the HD group (14.7 ± 1.3 pg/ml), only marginally increased in the MC group (21.9 ± 2.1 pg/ml), but highly and significantly elevated in the ICU group (186.1 ± 67.9 pg/ml, p<0.0001, **Tables 2**, **3**). Similarly, patients in the ICU group had significantly increased sIL-6R serum levels (26.2 ± 3.9 ng/ml, p<0.0001, **Figure 1B**, **Tables 2**, **3**) compared to the HD group (11.0 ± 1.2 ng/ml, **Figure 1B**). Importantly, sIL-6R levels were also significantly increased in the MC group (17.2 ± 1.5 ng/ml, **Figure 1B**), despite the fact that their COVID-19 infection had caused only mild symptoms and was several months ago. When we analyzed sgp130 serum levels in the same patient samples, no significant differences between the HD group (252.1 ± 5.6 ng/ml), the MC group (248.7 ± 6.2 ng/ml) and the ICU group (267.6 ± 9.8 ng/ml) were detected (**Figure 1C**). The significant main effects on group serum levels remain significant even after controlling for covariates (sex, age, BMI, quantity of previous illnesses). Only for sIL-6R age was a significant covariate (F(1, 139) = 6,738*), but had no impact on the main effect.

**Table 3:** Inter-group comparison for the different serum proteins.

	IL-6	IL-11	sIL-6R	sgp130	sCD25
ANOVA^§^	F(2, 139) = 14.49***	F(2, 139) = 1.14	F(2, 139) = 11.99***	F(2, 139) = 1.49	F(2, 139) = 40.43***
HD vs. MC	t(139) = -,271	t(139) =1,300	t(139) =-2,616	t(139) =,377	t(139) =,951
HD vs. ICU	t(139) = -4.94***	t(139) = 1,263	t(139) = -4,871***	t(139) = -1,341	t(139) = -7,934***
MC vs. ICU	t(139) = -4,973***	t(139) =,286	t(139) = -3,022*	t(139) = -1,711	t(139) = -8,560***

Multivariate ANVOA and intergroup comparisons for IL-6, IL-11, sIL-6R, sgp130 and sCD25 levels in healthy unexposed (HD) vs. mild COVID-19 convalescent (MC), HD vs. acute severe COVID-19 patients (ICU) and MC vs. ICU. ^§^Only for sIL-6R, age is a significant covariate (F(1, 139) = 6,738*). None of the other covariates (sex, age, BMI, number of known pre-existing conditions) had a significant impact on the main effect of group on serum levels. *p < 0.05 (two-tailed), ***p < 0.001 (two-tailed).

Previous studies reported both increased and decreased sgp130 levels in severe COVID-19 patients (42, 52). In summary, we find that IL-6 and sIL-6R serum levels are significantly increased in acute severe COVID-19 patients, and that increased sIL-6R levels can also be detected in mild COVID-19 convalescents several months after their infection, suggesting a long-term effect on sIL-6R generation, and thus IL-6 function due to the altered buffer, in these patients.

### Part of the IL-6 in acute severe COVID-19 patients is inactivated in complexes with sIL-6R and sgp130

**Figure 2:**
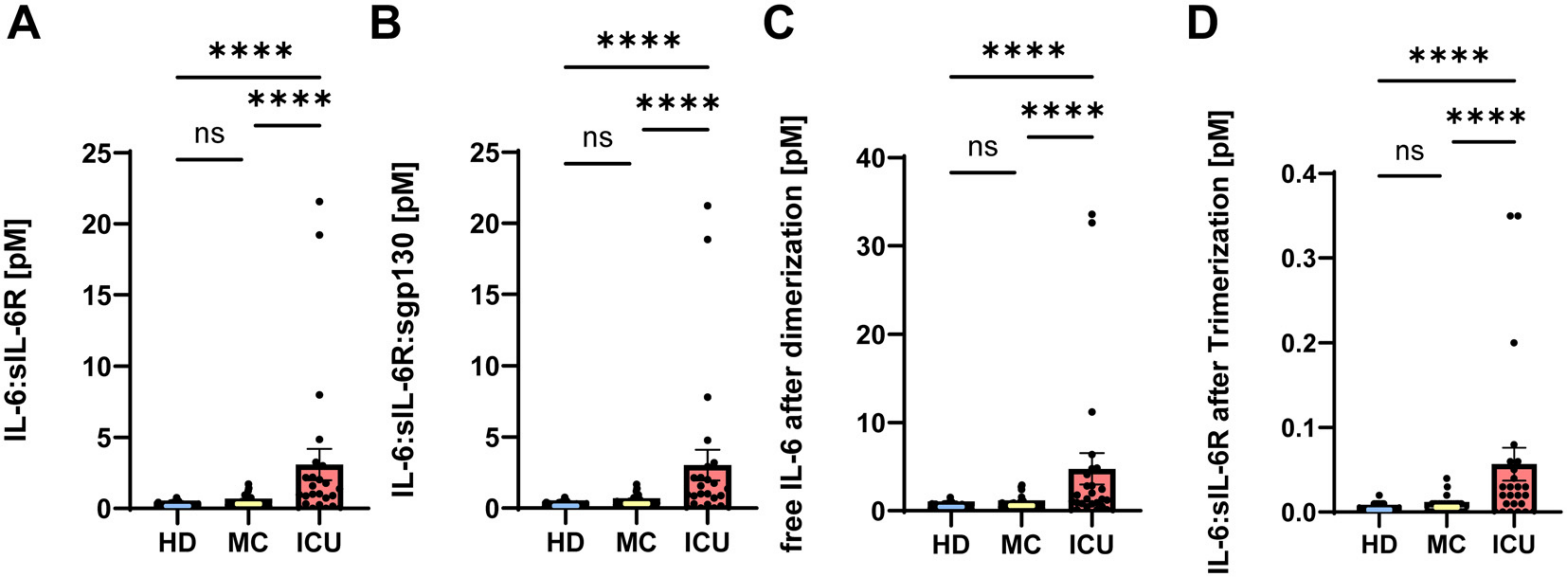
Part of the IL-6 in acute severe COVID-19 patients is trapped in inactive complexes. **(A, B)** Complexes of **(A)** IL-6:sIL-6R and **(B)** IL-6:sIL-6R:sgp130 were calculated based on the picomolar values shown in **Table 2** as described in Materials and Methods. The value of each individual is indicated, the mean is shown by a bar graph, and the error bars denote SEM. **(C)** Based on the amount of IL-6 that is inactivated in the IL-6:sIL-6R and IL-6:sIL-6R:sgp130 complexes, we calculated how much of the initial amounts of IL-6 was still free and not neutralized by complex formation. The value of each individual is indicated, the mean is shown by a bar graph and the error bars denote SEM. **(D)** Based on the amount of the IL-6:sIL-6R:sgp130 complexes and the dissociation constant, we calculated how much free IL-6:sIL-6R complexes are available that will not be neutralized by sgp130. The value of each individual is indicated, the mean is shown by a bar graph and the error bars denote SEM. Data were analyzed using one-way ANOVA following Tukey *post-hoc* test. The p values are shown above the respective diagrams as follows: ****p < 0.0001. n.s. denotes no significant difference.

Having shown that IL-6 and sIL-6R serum levels are increased in acute severe COVID-19 patients, we sought to determine how much of the IL-6 is trapped in inactive complexes and how much is free and able to do harmful activities, thereby potentially contributing to COVID-19 pathology. IL-6 binds to the sIL-6R with an affinity of 500 pM (46), and the resulting IL-6/sIL-6R complex binds to sgp130 with a higher affinity of 50 pM (47, 48). Whereas the IL-6:sIL-6R complex acts as an agonist and constitutes the pro-inflammatory part of the IL-6 biology (termed IL-6 trans-signaling) (17), the tripartite complex IL-6:sIL-6R:sgp130 is inactive, as it can no longer bind to cells in the body. Thus, not the pure IL-6 and sIL-6R levels are important to determine the possible impact of IL-6 and sIL-6R in a given disease, but rather how much active IL-6:sIL-6R complex and how much free IL-6 is present in the serum of a patient. In order to calculate this, we converted the amounts of IL-6, sIL-6R and sgp130 from ng/ml into pM (**Table 1**) and calculated first how much IL-6:sIL-6R complex can be found in the patients. As shown in **Figure 2A**, unexposed healthy controls had 0.17 ± 0.03 pM IL-6:sIL-6R complexes in their serum. Serum levels of patients IL-6:sIL-6R complexes in the MC group were higher (0.33 ± 0.04 pM), but this difference was not statistically significant. In contrast, IL-6:sIL-6R complex levels were significantly higher in ICU patients (3.09 ± 1.1 pM, p<0.001, **Figure 2A**). This is caused by the increase in both IL-6 and sIL-6R in these patients. Additionally, sIL-6R levels are always in molar excess over IL-6 levels, and thus an increase in IL-6, which we have seen in the ICU patients (**Figure 1A**), is the major driver of complex formation. Afterwards, we determined the amounts of the trimeric IL-6:sIL-6R:sgp130 complexes. As sgp130 levels are equal in all three groups (**Figure 1C**), the tripartite complex follows the same pattern with significantly elevated levels in ICU patients (3.04 ± 1.08 pM, p<0.001) compared to healthy controls (0.17 ± 0.02 pM) and mild convalescent patients (0.32 ± 0.04 pM, **Figure 2B**). Importantly, after calculation of the formed complexes, the free IL-6 levels were still significantly elevated in the ICU patients (4.77 ± 1.78 pM, p<0.0001) compared to healthy controls (0.45 ± 0.04 pM) and the mild convalescents (0.59 ± 0.06 pM, **Figure 2C**). Similarly, trans-signaling competent IL-6:sIL-6R complexes were still present after formation of the inactive tripartite complexes, which were significantly increased in ICU patients (0.06 ± 0.02 pM, p<0.0001) compared to healthy controls (0.003 ± 0.001 pM) and mild convalescents (0.006 ± 0.001 pM, **Figure 2D**). In summary, our results show that ICU patients on the one hand have more IL-6 that is inactivated in IL-6:sIL-6R:sgp130 complexes compared to the other two groups, but on the other hand have still significantly more free IL-6 and biologically active IL-6:sIL-6R complexes than healthy controls and mild convalescents.

### Analysis of immune cell subsets that are involved in IL-6 signaling during COVID-19 infection

**Figure 3:**
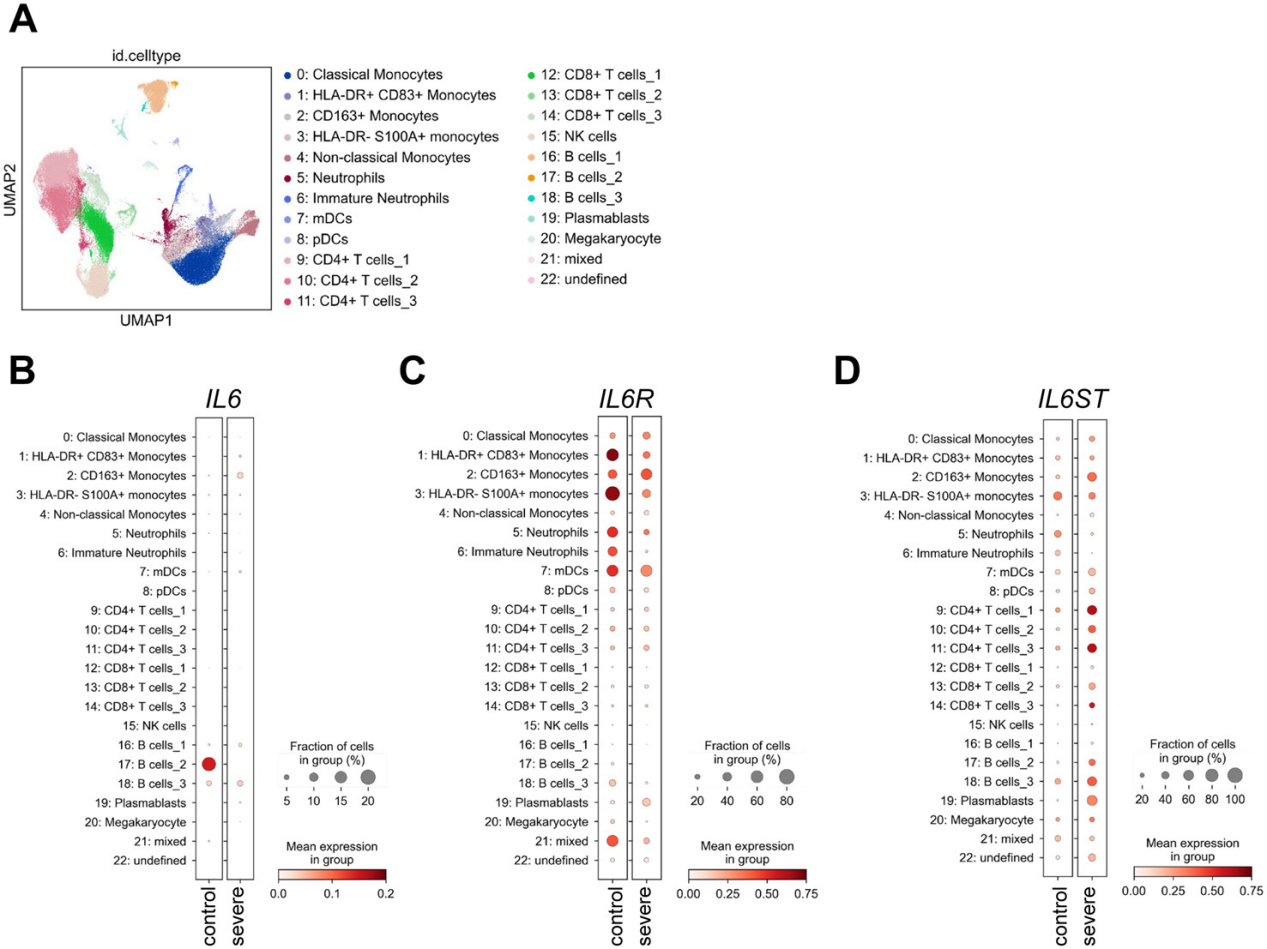
Expression of *IL6*, *IL6R* and *IL6ST* in uninfected controls and severe COVID-19 patients. **(A)** UMAP visualization of scRNA-seq profiles of PBMCs from 49 COVID-19 samples and 22 control samples colored according to cell type classification (Louvain clustering), reference-based cell-type annotation, and marker gene expression as described previously (49). **(B-D)** Expression of **(B)** *IL6*, **(C)** *IL6R* and **(D)** *IL6ST* in the 23 cell populations illustrated in panel **(A)**.

Cytokines can only act on cells that express the required receptors on their cell surface. Despite this fact, the question which cell types express which cytokine receptors is still largely unexplored, and whether the expression pattern of cytokine receptors is altered during disease states is also unclear (24). In order to obtain insights into this question, we used public single cell RNA sequencing data of immune cells derived from peripheral blood from severe COVID 19 patients and healthy controls published previously (49). We used the same strategy to identify different immune cell subsets as the original authors and analyzed gene expression in 23 distinguishable immune cell subsets (**Figure 3A**). Intriguingly, with the exception of one B cell subset in the healthy controls, immune cells did not significantly express *IL6*, which fits to the assumption that immune cells are not the only source of IL-6 production [**Figure 3B** and (53)]. *IL6R* expression, in contrast, was detected in monocytes, neutrophils and dendritic cells, whereas expression in T and B cells was less pronounced. Moreover, differential gene expression analysis revealed that *IL6R* expression was significantly increased in total monocytes and reduced in neutrophils of COVID-19 patients (**Figure 3C**; **Supplementary Figure S1**). *IL6ST*, which encodes the signal-transducing receptor gp130, was more uniformly expressed throughout the investigated cell types and its expression was significantly downregulated in neutrophils and upregulated in monocytes, CD4+ and CD8+ T cells and B cells from severe COVID-19 patients (**Figure 3D**; **Supplementary Figure S1**).

### No major role for systemic IL-11 in COVID-19 patients

**Figure 4:**
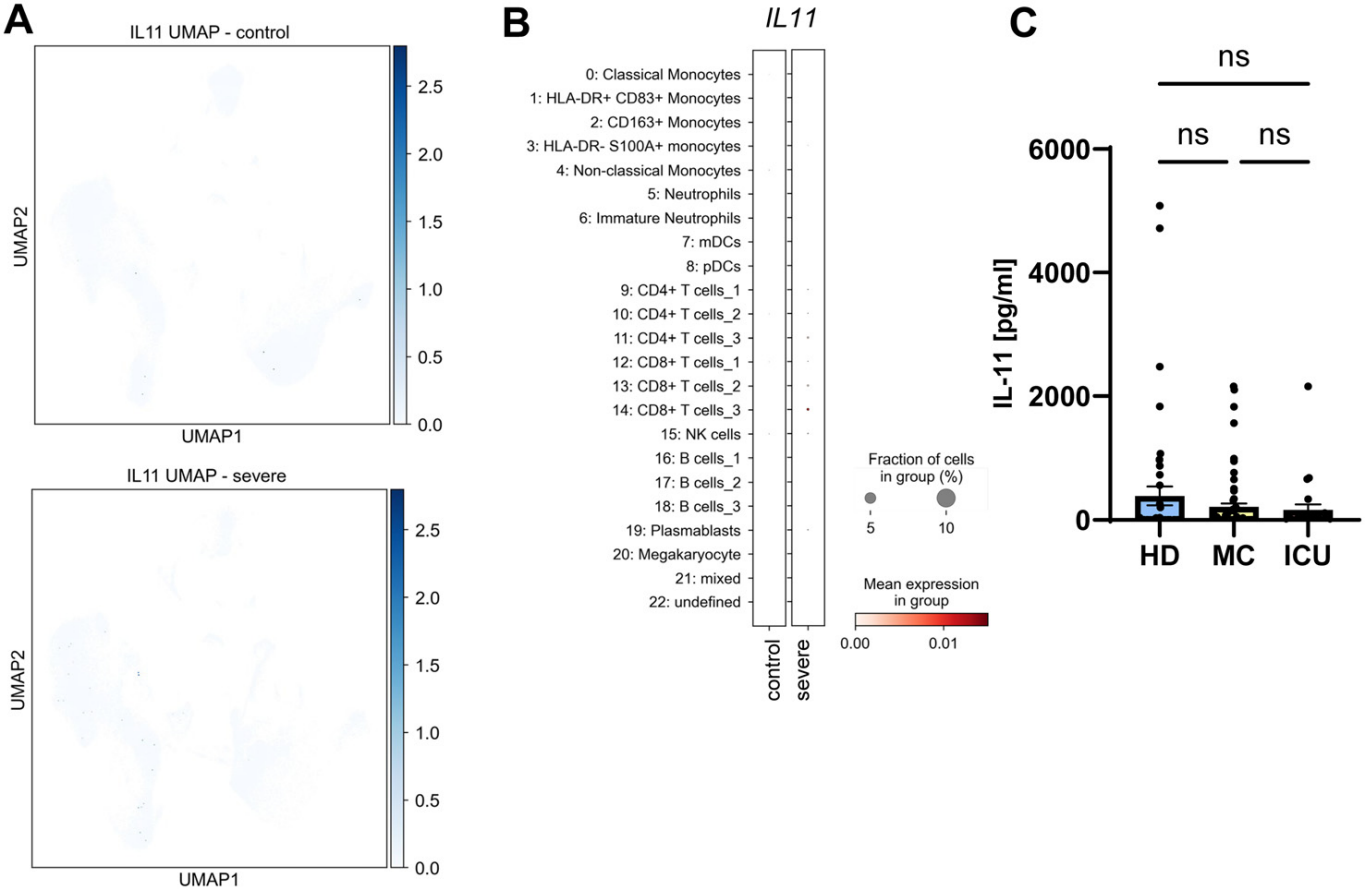
No major role for systemic IL-11 in COVID-19 infection. **(A)** UMAP visualization of scRNA-seq profiles of PBMCs from controls (upper panel) and severe COVID-19 patients (lower panel) for *IL11* expression. **(B)** Expression of *IL11* in the 23 different cell populations. scRNA-seq have been published previously (49). Please note that the low visibility of the dots is intended and reflects the low expression of *IL11* compared to the proteins analyzed in **Figures 3**, **5**. **(C)** Levels of IL-11 were determined by ELISA in the serum samples of 49 healthy unexposed individuals (HD), 68 mild COVID-19 convalescent (MC) and 25 acute severe COVID-19 patients (ICU). Serum amounts of each individual are shown as dots. The mean is indicated by bar graph, and the error bars denote SEM. Data were analyzed using one-way ANOVA following Tukey *post-hoc* test. The p values are shown above the respective diagrams as follows: n.s. denotes no significant difference.

Having found that IL-6 and sIL-6R serum levels are increased in acute severe COVID-19 patients, we sought to investigate whether this is a specific effect or whether similar proteins are also increased in the serum of these patients. IL-11 is the closest related protein to IL-6, as it belongs to the same cytokine family (12). IL-11 binds to a unique non-signaling IL-11 receptor (IL-11R) before it, like IL-6, recruits a homodimer of gp130 for signaling. Furthermore, previous work had shown that several respiratory viruses, e.g. respiratory syncytial virus (RSV), parainfluenza virus type 3 (PIV3) and rhinovirus (RV) 14 were potent inducers of IL-11 (54). In order to investigate whether COVID-19 infection would result in increased IL-11 levels, we first analyzed the above mentioned single cell RNA sequencing dataset. Intriguingly, *IL11* expression was very low or completely absent in healthy and diseased individuals (**Figure 4A**), which is also reflected by the very small expression dots of the individual cell types (**Figure 4B**). When we used a specific ELISA to detect IL-11 in the same serum samples we had analyzed previously, we found no significant differences between IL-11 serum levels in patients from the HD group (389.3 ± 151.5 pg/ml), the MC group (210.7 ± 58.8 pg/ml) and the ICU group (161.7 ± 91.6 pg/ml) (**Figure 4C**; **Tables 2**, **3**). These findings rule out a major role of IL-11 in COVID-19 infection and underline that induction of IL-11 is not a uniform cellular response to viral infection.

### Serum levels of sCD25 are increased in severe COVID-19 patients

**Figure 5:**
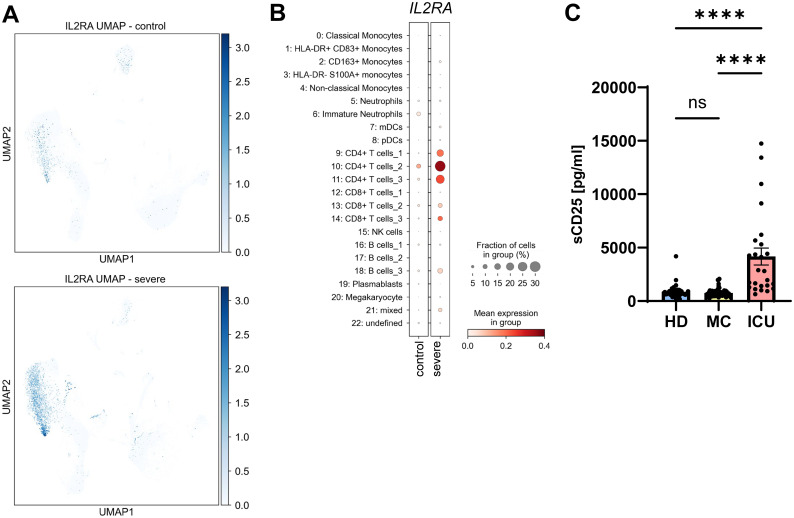
Serum levels of sCD25 are increased in severe COVID-19 patients. **(A)** UMAP visualization of scRNA-seq profiles of PBMCs from controls (upper panel) and severe COVID-19 patients (lower panel) for *CD25* expression. **(B)** Expression of *CD25* in the 23 different cell populations. scRNA-seq have been published previously (49). **(C)** Levels of sCD25 were determined by ELISA in the serum samples of 49 healthy unexposed individuals (HD), 68 mild COVID-19 convalescent (MC) and 25 acute severe COVID-19 patients (ICU). Serum amounts of each individual are shown as dots. The mean is indicated by bar graph, and the error bars denote SEM. Data were analyzed using one-way ANOVA following Tukey *post-hoc* test. The p values are shown above the respective diagrams as follows: ****p < 0.0001. n.s. denotes no significant difference.

We have recently shown that the soluble form of CD25 (sCD25) is generated by proteolytic cleavage of the membrane-bound CD25 by the metalloproteases ADAM10 and ADAM17 (29). Because the same proteases are responsible for the majority of the sIL-6R found in human serum (18), we investigated *CD25* expression and sCD25 levels in our cohorts. While *CD25* expression in healthy individuals was rather low and only moderately detectable in a distinct cell population, the levels were strongly increased in severe COVID-19 patients, being significantly upregulated in CD4+ T cells [**Figure 5A**; **Supplementary Figure S1**, (55)]. We further mapped the increased expression to one B cell cluster (**Figure 5B**). When we analyzed sCD25 levels via ELISA, there was no significant difference between the HD group (854.1 ± 82.7 pg/ml) and the MC group (765.4 ± 43.0 pg/ml, **Figure 5C**, **Tables 2**, **3**). However, sCD25 levels were significantly increased in serum samples from the ICU group compared to both the HD and the MC group, which is in good agreement with previous results [p < 0.0001, **Figure 3C**, **Tables 2**, **3**, (56)]. These results show that increased generation of soluble cytokine receptors in severe COVID-19 patients is not restricted to sIL-6R generation, but occurs for other cytokine receptors as well and might even be a general phenomenon. However, in contrast to sIL-6R (**Figure 1B**), we detected no increase in sCD25 in the MC group compared to healthy controls, which might be caused by different transcriptional and post-transcriptional mechanisms controlling expression and/or proteolysis of the two cytokine receptors.

### Determination of relevant correlations between disease state, serum proteins and possible confounders

Our data so far revealed differences for several serum proteins between healthy controls, mild convalescent and severely ill COVID-19 patients (**Table 3**). In order to determine how these data are correlated to possible confounders like age, sex, body mass index (BMI) or the number of known pre-existing conditions (**Table 1**), we coded the disease state (meaning whether the sample was from the HC, MC or ICU group) as one variable and performed a correlation analysis. We observed a positive correlation of the disease state with the age of the patients (r=0.33, p<0.01) and the number of known pre-existing conditions (r=0.42, p<0.01, **Table 4**). The numbers of known pre-existing conditions also highly correlated with the age (r=0.38, p<0.01) and the BMI of the patient (r=0.3, p<0.01, **Table 4**). These findings fit to previous studies showing that older age, higher body weight and known diseases increase the likelihood to experience a severe COVID-19 infection (6). We also noted significant correlations between the disease state and IL-6 serum levels (r=0.5, p<0.01), sIL-6R serum levels (r=0.33, p<0.01) and sCD25 levels (r=0.35, p<0.01), which is expected by our analysis shown in **Figures 1**, **2**. These correlations remain significant after controlling for the possible confounders (sex, age, BMI, quantity of previous illnesses). Intriguingly, the number of known pre-existing conditions was also highly correlated with IL-6 (r=0.25, p<0.01), sIL-6R (r=0.21, p<0.05) and sgp130 (r=0.29, p<0.01, **Table 4**) serum levels, underlining that pre-existing diseases are an important predictor of COVID-19 disease course.

**Table 4:** Determination of relevant correlations between disease state, serum proteins and/or possible confounders in all three groups.

	Group	Sex	Age	BMI	#PEC	IL-6	IL-11	sIL-6R	sgp130
Group	–	–	–	–	–	–	–	–	–
Sex^*^	-.18*	–	–	–	–	–	–	–	–
Age	.33**	-.28**	–	–	–	–	–	–	–
BMI^†^	.15	-.23**	.16	–	–	–	–	–	–
#PEC^‡^	.42**	.02	.38**	.30**	–	–	–	–	–
IL-6	.50**	-.07	.21*	.07	.25**	–	–	–	–
IL-11	-.02	.12	-.15	-.19*	-.12	.07	–	–	–
sIL-6R	.33**	-.13	.31**	.13	.21*	.14	-.12	–	–
sgp130	.09	-.25**	.35**	.03	.09	-.06	-.02	.34**	–
sCD25	.35**	-.13	.32**	.13	.29**	.27**	.20*	.12	.19*

Correlation analysis of disease state (group: 0 = HD, 1 = MC, 2 = ICU) with possible confounders and levels of serum proteins. ^*^0 = male, 1 = female; ^†^body mass index; ^‡^number of known pre-existing conditions. *p < 0.05 (two-tailed), **p < 0.01 (two-tailed).

Interestingly, serum levels of IL-6 (r=0.21, p<0.05), sIL-6R (r=0.31, p<0.01) and sgp130 (p=0.35, r<0.01, **Table 4**) were positively correlated with age, a correlation that we had previously not observed for these proteins in type 2 diabetes patients (27). Additionally, IL-11 was recently shown to be an important regulator for systemic adipogenesis (57), and the only significant factor that was correlated to IL-11 serum levels in our samples was the BMI (r=-0.19, p<0.05, **Table 4**). Importantly, sIL-6R levels were significantly correlated with sgp130 levels (r=0.34, p<0.01, **Table 4**), which we had also previously seen in healthy individuals as well as patients with type 2 diabetes and which led us to conclude that these two proteins constitute a natural occurring buffer system that is able to neutralize low amounts of IL-6 in the circulation (27).

### Determination of relevant correlations between serum proteins and possible confounders

Because it is challenging to determine correlations between three groups, we now analyzed COVID-19 patients (combining the MC and the ICU) and healthy controls separately regarding correlations with confounding factors. The number of known pre-existing conditions was still highly correlated with age (r=0.44, p<0.01) and BMI (r=0.33, r<0.01, **Table 5**) among people infected with COVID-19, but this was not the case for the healthy unexposed controls (**Table 5**). IL-6 serum levels in the patients correlated with age (r=0.29, p<0.01) and the number of known pre-existing conditions (r=0.26, p<0.05, **Table 5**), which was both not the case for the healthy unexposed controls, underlining that IL-6 levels are only relevant under pathological conditions (**Table 5**). We further observed significant correlations of IL-11 serum levels with sex (r=0.31, p<0.01) and inverse correlations with age (r=-0.34, p<0.01) and BMI (r=-0.24, p<0.05, **Table 5**). Again, such correlations were not detected in the healthy control samples (**Table 5**). In contrast, the correlation of sIL-6R serum levels with age was seen in patients (r=0.21, p<0.05) and even stronger in healthy controls (r=0.44, p<0.01, **Table 5**). Furthermore, sIL-6R serum levels significantly correlated with sgp130 levels, both in patients (r=0.3, p<0.01) and in healthy individuals (r=0.48, p<0.001, **Table 5**). Serum levels of sCD25 correlated significantly in COVID-19 patients with age (r=0.42, p<0.01), the number of known pre-existing conditions (r=0.37, p<0.01) and the IL-6 serum levels (r=0,29, p<0.01, **Table 5**). None of these correlations were detected in the healthy unexposed controls. In summary, these data clearly show that confounding factors that correlate with serum levels of different analyzed proteins can be differentiated between COVID-19 patients and healthy controls that have not been infected with SARS-CoV-2.

**Table 5:** Determination of relevant correlations between serum proteins and/or possible confounders.

	Sex	Age	BMI	#PEC	IL-6	IL-11	sIL-6R	sgp130	sCD25
Sex^*^	–	-.23*	-.12	.01	-.05	.31**	-.04	-.29**	-.20
Age	-.35*	–	.17	.44**	.29**	-.34**	.21*	.31**	.42**
BMI^†^	-.40**	.10	–	.33**	-.01	-.24*	.17	.12	.11
#PEC^‡^	.14	.11	.16	–	.26*	-.14	.18	.18	.37**
IL-6	.01	.02	.06	-.09	–	-.02	.03	-.07	.29**
IL-11	-.24	.20	-.14	-.13	.23	–	-.21*	-.14	.14
sIL-6R	-.22	.44**	-.02	.11	.08	.05	–	.30**	.18
sgp130	-.15	.45**	-.12	-.10	-.04	.20	.48***	–	.19
sCD25	.11	.09	.14	.03	.23	.30*	-.10	.14	–

Correlation analysis of COVID-19 patients (above diagonal, n = 99) and healthy unexposed controls (below diagonal, n = 49). ^*^0 = male, 1 = female; ^†^body mass index; ^‡^number of known pre-existing conditions. *p < 0.05 (two-tailed), **p < 0.01 (two-tailed), ***p < 0.001 (two-tailed).

### Determination of correlations between proteins and confounders in COVID-19 patients

Having shown these differences between patients and healthy controls, we next sought to determine whether correlations differed between mild COVID-19 convalescent (MC group) and acute severe COVID-19 patients (ICU group). In the MC group, the number of known pre-existing conditions was still significantly correlated with both the age (r=0.31, p<0.05) and the BMI (r=0.31, p<0.05, **Table 6**) of the patients, whereas in the ICU group only the BMI correlated with the pre-existing conditions (r=0.45, p<0.05, **Table 6**). Interestingly, the IL-11 serum levels in the MC group correlated significantly with sex (r=0.26, p<0.05) and were inversely correlated with age (r=-0.41, p<0.01) and BMI (r=-0.38, p<0.01, **Table 6**). A significant correlation between sIL-6R and sgp130 serum levels were detected in ICU patients (r=0.41, p<0.05, **Table 5**), but not in the convalescent patients (r=0.24, p>0.05, **Table 6**). Furthermore, IL-6 and sIL-6R serum levels were significantly inversely correlated in acute severe COVID-19 patients (r=-0.44, p<0.05, **Table 6**).

**Table 6:** Determination of relevant correlations between serum proteins and/or possible confounders only in COVID-19 patients.

	Sex	Age	BMI	#PEC	IL-6	IL-11	sIL-6R	sgp130	sCD25
Sex^*^	–	-.12	-.11	.19	.15	.26*	-.09	-.35**	-.11
Age	-.34	–	.27*	.31*	-.02	-.41**	.23	.28*	.06
BMI^†^	-.13	-.10	–	.31*	-.01	-.38**	.07	.16	.11
#PEC^‡^	-.24	-.12	.45*	–	.15	-.14	.11	.09	.05
IL-6	-.35	.37	-.14	-.34	–	.07	.01	-.10	-.09
IL-11	.41*	-.05	.21	-.01	-.07	–	-.31*	-.14	.27*
sIL-6R	.20	-.13	.38	.16	-.44*	.04	–	.24	.10
sgp130	-.05	.38	.06	.32	-.30	-.12	.41*	–	.13
sCD25	-.16	.48*	.06	.35	.04	.14	-.11	.40	–

Correlation analysis of mild COVID-19 convalescent (MC, above diagonal, n = 68) and acute severe COVID-19 patients (ICU, below diagonal, n = 25). ^*^0 = male, 1 = female; ^†^body mass index; ^‡^number of known pre-existing conditions. *p < 0.05 (two-tailed), **p < 0.01 (two-tailed).

In conclusion, our analysis shows that the serum profiles of mild convalescent and acute severe COVID-19 patients differ significantly.

## Discussion

The contribution of pro-inflammatory cytokines to the disease outcome in patients infected with SARS-CoV-2 has been identified within the first months of the pandemic, and especially the increased levels of IL-6 have been acknowledged to be of particular importance (58). IL-6 serves not only as a biomarker that is able to discriminate between patients with a mild and a severe disease course, but offers also an opportunity for therapeutic intervention. Accordingly, the first example of an effective treatment of severe COVID-19 patients through the blockade of IL-6R signaling with the monoclonal antibody tocilizumab was already published in May 2020 (59). However, IL-6 has multiple other functions despite its pro-inflammatory properties and contributes to tissue homeostasis and defense against pathogens, making IL-6 inhibition not a suitable approach for hospitalized patients in general, but rather only for severe cases (60).

In line with these previous findings, our cohorts showed increased levels of IL-6, especially in the ICU group (43). Furthermore, we observed increased sIL-6R levels not only in the ICU group, but also in the MC group, whereas sgp130 levels were not altered. We have previously postulated and shown that sIL-6R and sgp130 together form a buffer system that is able to bind and thus neutralize free circulating IL-6 (26, 27). The capacity of this buffer system is limited by the concentration of the sIL-6R, as sgp130 is always present in a molar excess compared to sIL-6R. The increased sIL-6R levels in the ICU group therefore increases the capacity of the buffer to neutralize IL-6. When we calculated the corresponding complexes accordingly, we found significantly more IL-6:sIL-6R complexes and significantly more IL-6:sIL-6R:sgp130 complexes, confirming that the increase in sIL-6R in the end resulted in more neutralized IL-6. However, due to the very high IL-6 levels in these patients, the buffer system is not capable of neutralizing all IL-6 molecules, and therefore there is still significantly more free IL-6 and biologically active IL-6:sIL-6R complexes in the ICU patients compared to the other groups, which is in line with clinical findings that especially in severe cases tocilizumab is an effective treatment (39, 40, 59). Thus, in contrast to our study on type 2 diabetes, in which the sIL-6R/sgp130 buffer system was disturbed (27), we observe no such effect in COVID-19 patients.

Our most important finding is a significant increase in sIL-6R in the MC group, which points to long lasting responses of the protease/cytokine receptor system even after the underlying SARS-CoV-2 infection has been resolved. Indeed, such an effect has also been seen for IL-22 in the same cohort (43). Importantly, this appears not a general phenomenon seen for all cytokine receptors, as we did not observe such a long lasting effect on sCD25, which is generated by the same proteases as the sIL-6R (29, 61). From our data, we cannot definitely determine the molecular mechanism behind this. We could not detect a major transcriptional up-regulation of the *IL6R* mRNA in immune cells from COVID-19 patients (**Supplementary Figure S1**), and as the major mechanism of sIL-6R generation is proteolysis (18), the increase in sIL-6R in both patient groups is therefore most likely due to enhanced proteolysis, which is in line with a previous study showing more IL-6R shedding induced by the SARS-CoV-2 spike protein (62). It is unclear which functional consequences the long-term elevated sIL-6R levels have in addition to the increased buffer capacity mentioned above. The single nucleotide polymorphism rs2228145, which results in the exchange of amino-acid residue Asp-358 to Ala-358 of the IL-6R and which makes the IL-6R more susceptible to proteolysis by the protease ADAM17 (63), results in increased sIL-6R serum levels in individuals which are homozygous for the minor allele (64). These individuals have reduced C-reactive protein concentrations and decreased odds of coronary heart disease events (65, 66). This example underlines the anti-inflammatory effect of increased sIL-6R levels.

We further observed an upregulation of *CD25* expression on CD4+ T cells from ICU patients along with increased sCD25 in the ICU group. Our previous data showed that enhanced expression of CD25 on T cells automatically results in more sCD25 due to cleavage (29), and this fits nicely to our data from ICU patients and is in accordance with previous work (67). How the differences between sIL-6R and sCD25 levels in the convalescent patients occur is currently unclear und requires further investigation.

In contrast to IL-6, we observed no increase in serum levels of IL-11 in COVID-19 patients. Interestingly, a recent study showed that expression of the proteins ORF6, ORF8, ORF9b or ORF9c from SARS-CoV-2 in A549 cells induces the expression of *IL11* and contributes to pro-fibrotic effects (68). This is not necessarily a contradiction, as cytokines are known to act locally and reach much higher concentrations at sites of infection or inflammation than in the general circulation as reflected in the serum levels that we analyzed in our study. Further studies using e.g. bronchoalveolar lavage or even tissue biopsies from patients would be able to determine whether IL-11 might be present in higher amounts locally in the lung tissue of COVID-19 patients and thereby contribute to inflammatory or pro-fibrotic processes.

Our study has limitations, especially the rather small number of participants in each group. However, we were able to replicate the influence of known confounders like age, sex, BMI and pre-existing medical conditions that have been identified in previous, larger studies, underlining that our cohorts studied here are suitable to draw solid conclusions. Furthermore, we were able to determine novel correlations between the different serum proteins investigated in this study that have not been determined previously and which will be helpful to design better therapeutic approaches targeting IL-6/IL-6R signaling than simply blocking all IL-6R using tocilizumab.

In conclusion, we provide evidence that an increase in sIL-6R levels is not only present in severely ill COVID-19 patients, but that this increase is also detectable in convalescent patients after a mild disease. This increase in sIL-6R results in more IL-6 that is neutralized in IL-6:sIL-6R:sgp130 complexes, but the high IL-6 levels in the ICU patients lead to an overload of the sIL-6R/sgp130 buffer system, resulting in still more free IL-6 and biologically active IL-6:sIL-6R complexes in ICU patients compared to MC and healthy controls.

## Data Availability

The original contributions presented in the study are included in the article/**Supplementary Material**. Further inquiries can be directed to the corresponding author.

## Supplementary material

Supplementary Figure 1 Differential gene expression analysis. **(A-E)** Shown are the log2-transformed fold-changes in relation to the negative log10-transformed adjusted p-values for IL6ST, IL6R and IL2RA in **(A)** monocytes, **(B)** neutrophils, **(C)** CD4+ T cells, **(D)** CD8+ T cells and **(E)** B cells. Genes above the dashed line are significantly regulated. scRNA-seq have been published previously (49).

## References

[1] Cascella M Rajnik M Aleem A Dulebohn SC Di Napoli R. Features, Evaluation, and Treatment of Coronavirus (COVID-19). In: StatPearls. Treasure Island (FL): StatPearls (2024). 32150360

[2] Markov PV Ghafari M Beer M Lythgoe K Simmonds P Stilianakis NI. The evolution of SARS-CoV-2. Nat Rev Microbiol. (2023) 21:361–79. doi: 10.1038/s41579-023-00878-2 37020110

[3] Statsenko Y Al Zahmi F Habuza T Almansoori TM Smetanina D Simiyu GL. Impact of age and sex on COVID-19 severity assessed from radiologic and clinical findings. Front Cell Infect Microbiol. (2021) 11:777070. doi: 10.3389/fcimb.2021.777070 35282595 PMC8913498

[4] Davies NG Klepac P Liu Y Prem K Jit M CMMID COVID-19 working group. Age-dependent effects in the transmission and control of COVID-19 epidemics. Nat Med. (2020) 26:1205–11. doi: 10.1038/s41591-020-0962-9 32546824

[5] Peng M He J Xue Y Yang X Liu S Gong Z. Role of hypertension on the severity of COVID-19: A review. J Cardiovasc Pharmacol. (2021) 78:e648–55. doi: 10.1097/FJC.0000000000001116 PMC8562915 34321401

[6] Kharroubi SA Diab-El-Harake M. Sex-differences in COVID-19 diagnosis, risk factors and disease comorbidities: A large US-based cohort study. Front Public Health. (2022) 10:1029190. doi: 10.3389/fpubh.2022.1029190 36466473 PMC9714345

[7] Leung JM Yang CX Tam A Shaipanich T Hackett T-L Singhera GK. ACE-2 expression in the small airway epithelia of smokers and COPD patients: implications for COVID-19. Eur Respir J. (2020) 55. doi: 10.1183/13993003.00688-2020 PMC7144263 32269089

[8] Sawadogo W Tsegaye M Gizaw A Adera T. Overweight and obesity as risk factors for COVID-19-associated hospitalisations and death: systematic review and meta-analysis. BMJ Nutr Prev Health. (2022) 5:10–8. doi: 10.1136/bmjnph-2021-000375 PMC8783972 35814718

[9] Guo W Li M Dong Y Zhou H Zhang Z Tian C. Diabetes is a risk factor for the progression and prognosis of COVID-19. Diabetes Metab Res Rev. (2020) 36:e3319. doi: 10.1002/dmrr.v36.7 32233013 PMC7228407

[10] Hasanvand A. COVID-19 and the role of cytokines in this disease. Inflammopharmacology. (2022) 30:789–98. doi: 10.1007/s10787-022-00992-2 PMC9064717 35505267

[11] Webb BJ Peltan ID Jensen P Hoda D Hunter B Silver A. Clinical criteria for COVID-19-associated hyperinflammatory syndrome: a cohort study. Lancet Rheumatol. (2020) 2:e754–63. doi: 10.1016/S2665-9913(20)30343-X PMC7524533 33015645

[12] Garbers C Hermanns HM Schaper F Müller-Newen G Grötzinger J Rose-John S. Plasticity and cross-talk of Interleukin 6-type cytokines. Cytokine Growth Factor Rev. (2012) 23:85–97. doi: 10.1016/j.cytogfr.2012.04.001 22595692

[13] Heinrich PC Behrmann I Haan S Hermanns HM Müller-Newen G Schaper F. Principles of interleukin (IL)-6-type cytokine signalling and its regulation. Biochem J. (2003) 374:1–20. doi: 10.1042/bj20030407 12773095 PMC1223585

[14] Garbers C Scheller J. Interleukin-6 and interleukin-11: same same but different. Biol Chem. (2013) 394:1145–61. doi: 10.1515/hsz-2013-0166 23740659

[15] Lokau J Agthe M Flynn CM Garbers C. Proteolytic control of Interleukin-11 and Interleukin-6 biology. Biochim Biophys Acta. (2017) 1864:2105–17. doi: 10.1016/j.bbamcr.2017.06.008 28630024

[16] Lokau J Agthe M Garbers C. Generation of soluble interleukin-11 and interleukin-6 receptors: A crucial function for proteases during inflammation. Mediators Inflammation. (2016) 2016:1785021. doi: 10.1155/2016/1785021 PMC4963573 27493449

[17] Rose-John S Jenkins BJ Garbers C Moll JM Scheller J. Targeting IL-6 trans-signalling: past, present and future prospects. Nat Rev Immunol. (2023) 23:666–81. doi: 10.1038/s41577-023-00856-y PMC10108826 37069261

[18] Riethmueller S Somasundaram P Ehlers JC Hung C-W Flynn CM Lokau J. Proteolytic origin of the soluble human IL-6R *in vivo* and a decisive role of N-glycosylation. PloS Biol. (2017) 15:e2000080. doi: 10.1371/journal.pbio.2000080 28060820 PMC5218472

[19] Lokau J Nitz R Agthe M Monhasery N Aparicio-Siegmund S Schumacher N. Proteolytic cleavage governs interleukin-11 trans-signaling. Cell Rep. (2016) 14:1761–73. doi: 10.1016/j.celrep.2016.01.053 26876177

[20] Koch L Kespohl B Agthe M Schumertl T Düsterhöft S Lemberg MK. Interleukin-11 (IL-11) receptor cleavage by the rhomboid protease RHBDL2 induces IL-11 trans-signaling. FASEB J. (2021) 35:e21380. doi: 10.1096/fj.202002087R 33566379 PMC12266321

[21] Chalaris A Garbers C Rabe B Rose-John S Scheller J. The soluble Interleukin 6 receptor: generation and role in inflammation and cancer. Eur J Cell Biol. (2011) 90:484–94. doi: 10.1016/j.ejcb.2010.10.007 21145125

[22] Sommer J Garbers C Wolf J Trad A Moll JM Sack M. Alternative intronic polyadenylation generates the interleukin-6 trans-signaling inhibitor sgp130-E10. J Biol Chem. (2014) 289:22140–50. doi: 10.1074/jbc.M114.560938 PMC4139227 24973212

[23] Wolf J Waetzig GH Chalaris A Reinheimer TM Wege H Rose-John S. Different soluble forms of the interleukin-6 family signal transducer gp130 fine-tune the blockade of interleukin-6 trans-signaling. J Biol Chem. (2016) 291:16186–96. doi: 10.1074/jbc.M116.718551 PMC4965567 27226573

[24] Wolf J Rose-John S Garbers C. Interleukin-6 and its receptors: a highly regulated and dynamic system. Cytokine. (2014) 70:11–20. doi: 10.1016/j.cyto.2014.05.024 24986424

[25] Muller SA Shmueli MD Feng X Tüshaus J Schumacher N Clark R. The Alzheimer’s disease-linked protease BACE1 modulates neuronal IL-6 signaling through shedding of the receptor gp130. Mol Neurodegener. (2023) 18:13. doi: 10.1186/s13024-023-00596-6 36810097 PMC9942414

[26] Garbers C Aparicio-Siegmund S Rose-John S. The IL-6/gp130/STAT3 signaling axis: recent advances towards specific inhibition. Curr Opin Immunol. (2015) 34:75–82. doi: 10.1016/j.coi.2015.02.008 25749511

[27] Aparicio-Siegmund S Garbers Y Flynn CM Waetzig GH Gouni-Berthold I Krone W. The IL-6-neutralizing sIL-6R-sgp130 buffer system is disturbed in patients with type 2 diabetes. Am J Physiol Endocrinol Metab. (2019) 317:E411–20. doi: 10.1152/ajpendo.00166.2019 31237452

[28] Lokau J Garbers C. Biological functions and therapeutic opportunities of soluble cytokine receptors. Cytokine Growth Factor Rev. (2020) 55:94–108. doi: 10.1016/j.cytogfr.2020.04.003 32386776

[29] Kirschke S Ogunsulire I Selvakumar B Schumacher N Sezin T Rose-John S. The metalloprotease ADAM10 generates soluble interleukin-2 receptor alpha (sCD25) *in vivo* . J Biol Chem. (2022) 298:101910. doi: 10.1016/j.jbc.2022.101910 35398356 PMC9127578

[30] Lokau J Petasch LM Garbers C. The soluble IL-2 receptor alpha/CD25 as a modulator of IL-2 function. Immunology. (2023) 3:377–8. doi: 10.1111/imm.13723 38037265

[31] Giraldez MD Carneros D Garbers C Rose-John S Bustos M. New insights into IL-6 family cytokines in metabolism, hepatology and gastroenterology. Nat Rev Gastroenterol Hepatol. (2021) 18:787–803. doi: 10.1038/s41575-021-00473-x 34211157

[32] Kang S Narazaki M Metwally H Kishimoto T. Historical overview of the interleukin-6 family cytokine. J Exp Med. (2020) 217:e20190347. doi: 10.1084/jem.20190347 32267936 PMC7201933

[33] Schumertl T Lokau J Rose-John S Garbers C. Function and proteolytic generation of the soluble interleukin-6 receptor in health and disease. Biochim Biophys Acta Mol Cell Res. (2022) 1869:119143. doi: 10.1016/j.bbamcr.2021.119143 34626681

[34] Garbers C Heink S Korn T Rose-John S. Interleukin-6: designing specific therapeutics for a complex cytokine. Nat Rev Drug Discovery. (2018) 17:395–412. doi: 10.1038/nrd.2018.45 29725131

[35] Schulte DM Waetzig GH Schuett H Marx M Schulte B Garbers C. Case report: arterial wall inflammation in atherosclerotic cardiovascular disease is reduced by olamkicept (sgp130Fc). Front Pharmacol. (2022) 13:758233. doi: 10.3389/fphar.2022.758233 35754497 PMC9218605

[36] Schreiber S Aden K Bernardes JP Conrad C Tran F Höper H. Therapeutic IL-6 trans-signalling inhibition by olamkicept (sgp130Fc) in patients with active inflammatory bowel disease. Gastroenterology. (2021) 160:2354–2366.e11. doi: 10.1053/j.gastro.2021.02.062 33667488

[37] Del Valle DM Kim-Schulze S Huang H-H Beckmann ND Nirenberg S Wang B. An inflammatory cytokine signature predicts COVID-19 severity and survival. Nat Med. (2020) 26:1636–43. doi: 10.1038/s41591-020-1051-9 PMC7869028 32839624

[38] Piscoya A Parra Del Riego A Cerna-Viacava R Rocco J Roman YM Escobedo AA. Efficacy and harms of tocilizumab for the treatment of COVID-19 patients: A systematic review and meta-analysis. PloS One. (2022) 17:e0269368. doi: 10.1371/journal.pone.0269368 35657993 PMC9165853

[39] Chilimuri S Sun H Alemam A Kang K-S Lao P Mantri N. Tocilizumab use in patients with moderate to severe COVID-19: A retrospective cohort study. J Clin Pharm Ther. (2021) 46:440–6. doi: 10.1111/jcpt.13303 33098139

[40] Eimer J Vesterbacka J Svensson A-K Stojanovic B Wagrell C Sönnerborg A. Tocilizumab shortens time on mechanical ventilation and length of hospital stay in patients with severe COVID-19: a retrospective cohort study. J Intern Med. (2021) 289:434–6. doi: 10.1111/joim.v289.3 PMC7436415 32744399

[41] Group RC. Tocilizumab in patients admitted to hospital with COVID-19 (RECOVERY): a randomised, controlled, open-label, platform trial. Lancet. (2021) 397:1637–45. doi: 10.1016/S0140-6736(21)00676-0 PMC8084355 33933206

[42] Rodriguez-Hernandez MA Carneros D Núñez- Núñez M Coca R Baena R López-Ruiz G. Identification of IL-6 signalling components as predictors of severity and outcome in COVID-19. Front Immunol. (2022) 13:891456. doi: 10.3389/fimmu.2022.891456 35634332 PMC9137400

[43] Meltendorf S Vogel K Thurm C Prätsch F Reinhold A Färber J. IL-13 determines specific IgE responses and SARS-CoV-2 immunity after mild COVID-19 and novel mRNA vaccination. Eur J Immunol. (2022) 52:1972–9. doi: 10.1002/eji.202249951 PMC9874813 36271745

[44] Lingel H Meltendorf S Billing U Thurm C Vogel K Majer C. Unique autoantibody prevalence in long-term recovered SARS-CoV-2-infected individuals. J Autoimmun. (2021) 122:102682. doi: 10.1016/j.jaut.2021.102682 34214763 PMC8214939

[45] Konietschke F Bösiger S Brunner E Hothorn LA. Are multiple contrast tests superior to the ANOVA? Int J Biostat. (2013) 9(1). doi: 10.1515/ijb-2012-0020 23902695

[46] Weiergräber O Hemmann U Küster A Müller-Newen G Schneider J Rose-John S. Soluble human interleukin-6 receptor. Expression in insect cells, purification and characterization. Eur J Biochem. (1995) 234:661–9. doi: 10.1111/j.1432-1033.1995.661_b.x 8536717

[47] Hibi M Murakami M Saito M Hirano T Taga T Kishimoto T. Molecular cloning and expression of an IL-6 signal transducer, gp130. Cell. (1990) 63:1149–57. doi: 10.1016/0092-8674(90)90411-7 2261637

[48] Zohlnhöfer D Graeve L Rose-John S Schooltink H Heinrich PC. The hepatic interleukin-6 receptor. Down-regulation of the interleukin-6 binding subunit (gp80) by its ligand. FEBS Lett. (1992) 306:219–22. doi: 10.1016/0014-5793(92)81004-65 1321736

[49] Schulte-Schrepping J Reusch N Paclik D Baßler K Schlickeiser S Zhang B. Severe COVID-19 is marked by a dysregulated myeloid cell compartment. Cell. (2020) 182:1419–1440 e23. doi: 10.1016/j.cell.2020.08.001 32810438 PMC7405822

[50] Wolf FA Angerer P Theis FJ. SCANPY: large-scale single-cell gene expression data analysis. Genome Biol. (2018) 19:15. doi: 10.1186/s13059-017-1382-0 29409532 PMC5802054

[51] Nikolaus S Waetzig GH Butzin S Ziolkiewicz M Al-Massad N Thieme F. Evaluation of interleukin-6 and its soluble receptor components sIL-6R and sgp130 as markers of inflammation in inflammatory bowel diseases. Int J Colorectal Dis. (2018) 33:927–36. doi: 10.1007/s00384-018-3069-8 PMC6002455 29748708

[52] Di Spigna G Covelli B Vargas M Di Caprio R Rubino V Iacovazzo C. The behaviour of IL-6 and its soluble receptor complex during different waves of the COVID-19 pandemic. Life (Basel). (2024) 14. doi: 10.3390/life14070814 PMC11278279 39063569

[53] Narazaki M Kishimoto T. Current status and prospects of IL-6-targeting therapy. Expert Rev Clin Pharmacol. (2022) 15:575–92. doi: 10.1080/17512433.2022.2097905 35791866

[54] Einarsson O Geba GP Zhu Z Landry M Elias JA. Interleukin-11: stimulation *in vivo* and *in vitro* by respiratory viruses and induction of airways hyperresponsiveness. J Clin Invest. (1996) 97:915–24. doi: 10.1172/JCI118514 PMC507136 8613544

[55] Ryffel B Willcocks JL Brooks N Woerly G. Interleukin-2 receptor (CD25) upregulation on human T-lymphocytes: sensitivity to immunosuppressants is defined by the mode of T-lymphocyte activation. Immunopharmacology. (1995) 30:199–207. doi: 10.1016/0162-3109(95)00023-M 8557519

[56] Xie M Yunis J Yao Y Shi J Yang Y Zhou P. High levels of soluble CD25 in COVID-19 severity suggest a divergence between anti-viral and pro-inflammatory T-cell responses. Clin Transl Immunol. (2021) 10:e1251. doi: 10.1002/cti2.v10.2 PMC7883478 33614032

[57] Dong B Hiasa M Higa Y Ohnishi Y Endo I Kondo T. Osteoblast/osteocyte-derived interleukin-11 regulates osteogenesis and systemic adipogenesis. Nat Commun. (2022) 13:7194. doi: 10.1038/s41467-022-34869-3 36424386 PMC9691688

[58] Coomes EA Haghbayan H. Interleukin-6 in Covid-19: A systematic review and meta-analysis. Rev Med Virol. (2020) 30:1–9. doi: 10.1002/rmv.v30.6 PMC7460877 32845568

[59] Xu X Han M Li T Sun W Wang D Fu B. Effective treatment of severe COVID-19 patients with tocilizumab. Proc Natl Acad Sci U.S.A. (2020) 117:10970–5. doi: 10.1073/pnas.2005615117 PMC7245089 32350134

[60] Jones SA Hunter CA. Is IL-6 a key cytokine target for therapy in COVID-19? Nat Rev Immunol. (2021) 21:337–9. doi: 10.1038/s41577-021-00553-8 PMC8043092 33850327

[61] Garbers C Jänner N Chalaris A Moss ML Floss DM Meyer D. Species specificity of ADAM10 and ADAM17 proteins in interleukin-6 (IL-6) trans-signaling and novel role of ADAM10 in inducible IL-6 receptor shedding. J Biol Chem. (2011) 286:14804–11. doi: 10.1074/jbc.M111.229393 PMC3083187 21454673

[62] Patra T Meyer K Geerling L Isbell TS Hoft DF Brien J. SARS-CoV-2 spike protein promotes IL-6 trans-signaling by activation of angiotensin II receptor signaling in epithelial cells. PloS Pathog. (2020) 16:e1009128. doi: 10.1371/journal.ppat.1009128 33284859 PMC7746263

[63] Garbers C Monhasery N Aparicio-Siegmund S Lokau J Baran P Nowell MA. The interleukin-6 receptor asp358Ala single nucleotide polymorphism rs2228145 confers increased proteolytic conversion rates by ADAM proteases. Biochim Biophys Acta. (2014) 1842:1485–94. doi: 10.1016/j.bbadis.2014.05.018 24878322

[64] Rafiq S Frayling TM Murray A Hurst A Stevens K Weedon MN. A common variant of the interleukin 6 receptor (IL-6r) gene increases IL-6r and IL-6 levels, without other inflammatory effects. Genes Immun. (2007) 8:552–9. doi: 10.1038/sj.gene.6364414 PMC2668154 17671508

[65] Sarwar N Butterworth AS Freitag DF Gregson J Willeit P Gorman DN. Interleukin-6 receptor pathways in coronary heart disease: a collaborative meta-analysis of 82 studies. Lancet. (2012) 379:1205–13. doi: 10.1016/S0140-6736(11)61931-4 PMC3316940 22421339

[66] Swerdlow D Holmes MV Kuchenbaecker KB Engmann JEL Shah T Sofat R. The interleukin-6 receptor as a target for prevention of coronary heart disease: a mendelian randomisation analysis. Lancet. (2012) 379:1214–24. doi: 10.1016/S0140-6736(12)60110-X PMC3316968 22421340

[67] Monserrat J Gómez-Lahoz A Ortega MA Sanz J Muñoz B Arévalo-Serrano J. Role of innate and adaptive cytokines in the survival of COVID-19 patients. Int J Mol Sci. (2022) 23. doi: 10.3390/ijms231810344 PMC9499609 36142255

[68] Lopez-Ayllon BD de Lucas-Rius A Mendoza-García L García-García T Fernández-Rodríguez R Suárez-Cárdenas J. SARS-CoV-2 accessory proteins involvement in inflammatory and profibrotic processes through IL11 signaling. Front Immunol. (2023) 14:1220306. doi: 10.3389/fimmu.2023.1220306 37545510 PMC10399023

