# Supplementary figures and images for: Long-term increase in soluble interleukin-6 receptor levels in convalescents after mild COVID-19 infection
Source: Front Immunol. 2025 Jan 6;15:1488745. doi: 10.3389/fimmu.2024.1488745 (PMC11743636; doi:10.3389/fimmu.2024.1488745)

Supplemental Figure 1

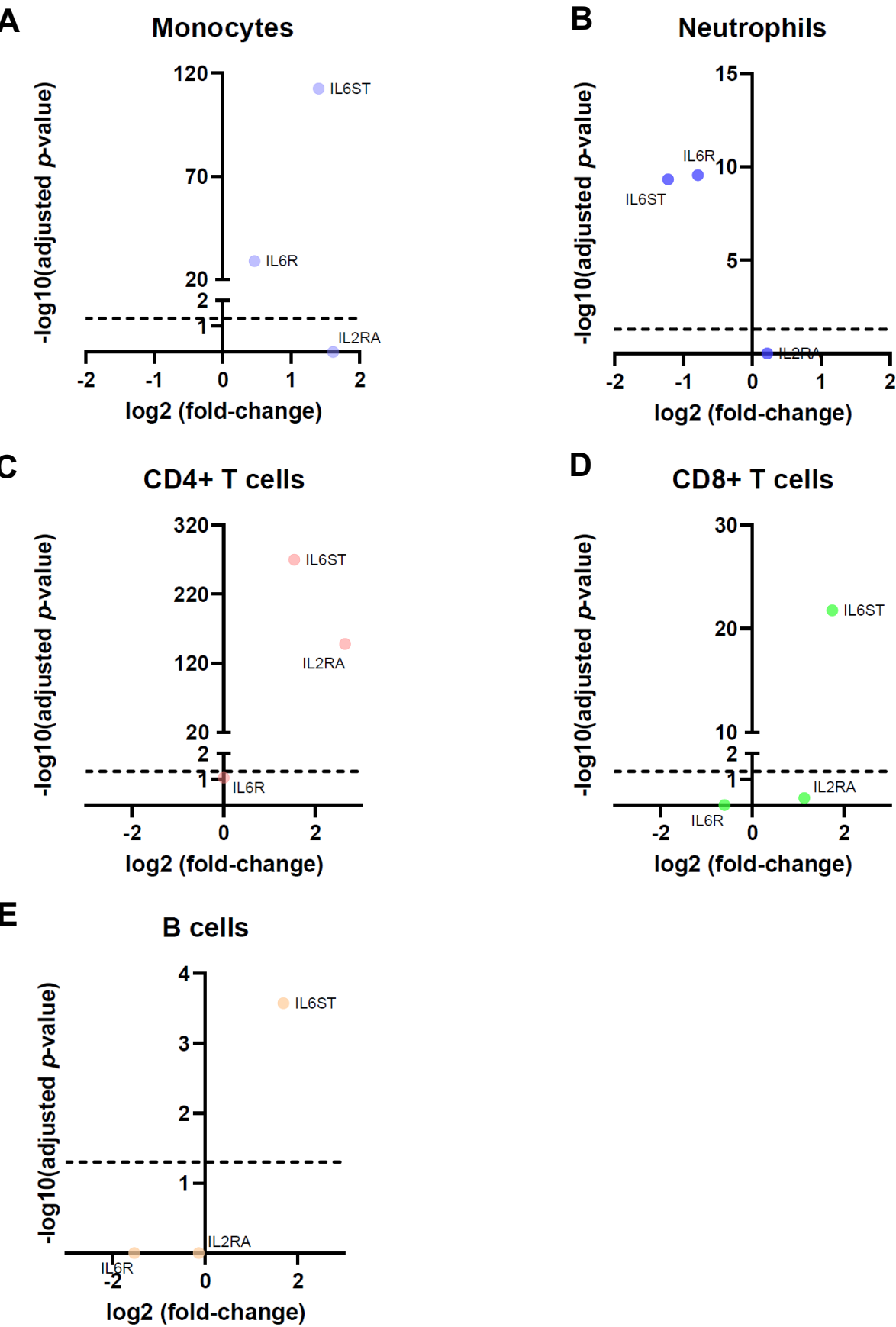

Supplement: Supplementary Figure 1 — Differential gene expression analysis. (A-E) Shown are the log2-transformed fold-changes in relation to the negative log10-transformed adjusted p-values for IL6ST, IL6R and IL2RA in (A) monocytes, (B) neutrophils, (C) CD4+ T cells, (D) CD8+ T cells and (E) B cells. Genes above the dashed line are significantly regulated. scRNA-seq have been published previously (49). [file DataSheet1.pdf]
